# Influence of climatic and land use factors on post-monsoon distribution of *Aedes* mosquito vectors in Udupi taluk

**DOI:** 10.1038/s41598-025-20413-y

**Published:** 2025-10-21

**Authors:** Prathiksha Prakash Nayak, Jagadeesha Pai B, Sreejith Govindan, Naren Babu N

**Affiliations:** 1 Department of Civil Engineering, Manipal Institute of Technology, Manipal Academy of Higher Education, Manipal, India 576104,; 2 Division of Microbiology, Department of Basic Medical Sciences, Manipal Academy of Higher Education, Manipal, India 576104,; 3 Manipal Institute of Virology, Manipal Academy of Higher Education, Manipal, India 576104,

**Keywords:** *Aedes* mosquitoes, GIS, Climatic factors, Vector-Borne diseases, Climate sciences, Environmental sciences, Diseases

## Abstract

Vector-borne diseases (VBDs) account for 17% of the global disease burden, causing approximately 700,000 deaths annually. Among these, dengue is a major public health concern in tropical and subtropical regions, transmitted primarily by *Aedes aegypti* and *Aedes albopictus*. This cross-sectional observational study was conducted in Udupi taluk, Karnataka, India, to examine the influence of climatic factors and land use/land cover (LULC) on *Aedes* distribution during the post-monsoon season. With a 5 km × 5 km grid sampling design, 28 locations (560 households) were surveyed between October and December 2023. *Aedes* larvae were detected in 71.42% of houses. Of the 425 mosquitoes identified, *A. albopictus* (57.64%) were more abundant than *A. aegypti* (23.52%), with the rest belonging to *Culex* and *Armigeres.* Climatic factors such as temperature and humidity showed positive correlation with *A.albopictus*, while *A.aegypti* showed weak negative correlation. GIS analysis highlighted the *Aedes* high-density breeding areas, and larval indices maps were developed. Zero-Inflated Negative Binomial (ZINB) regression identified HI as a significant predictor of *A. aegypti* abundance. LULC mapping indicated *Aedes* breeding activity concentrated in fragmented urban areas and croplands. The study highlights the importance of integrating climatic and spatial data for targeted vector control.

**Supplementary Information:**

The online version contains supplementary material available at 10.1038/s41598-025-20413-y.

## Introduction

According to the World Health Organisation (WHO), vector-borne infections account for 17% of the global disease burden, resulting in 700,000 fatalities annually. Among the Vector-borne diseases (VBDs), mosquito-borne infections like malaria and dengue are estimated to be 219 million and 96 million cases, respectively, across the globe annually^1^. Mosquitoes that carry infectious agents can transmit the infection for their entire lives under favourable conditions^1–3^. *Aedes* mosquito species transmit arboviral diseases like dengue, chikungunya, Zika, and yellow fever^4^. These infections are present in tropics and subtropical regions due to the presence of abundant mosquito vectors, favourable climatic conditions, population movement, and urbanisation^1,5,6^. Dengue is an important disease with high morbidity and mortality among all the *Aedes*-borne infections^5–9^. Due to the absence of a universally effective vaccine and specific antiviral treatments for dengue and chikungunya, disease management remains largely supportive. The limited efficacy and restricted use of existing vaccines further constrain prevention efforts. Therefore, vector control serves as the primary intervention to interrupt *Aedes*-borne virus transmission and remains central to outbreak prevention strategies^6,10–12^.

*Aedes aegypti* and *Aedes albopictus* are the most prevalent species that transmit dengue. *Aedes* species larval breeding occurs in urban areas in water-holding containers in intra-domestic as well as peridomestic premises of houses, in fridge trays, flower vases, rubber tyres, coconut shells, plastic containers, flowerpots, etc^13^. The destruction of such breeding habitats would reduce the number of disease cases and transmission rate^14^. *Aedes* species are believed to be sensitive to changes in climatic factors, growing populations, and urbanisation^15,16^. These species are closely associated with humans, making them a significant vector of human pathogens. Since 1950, *A. aegypti’s* environmental suitability has increased globally by up to 1.5% per decade, and this trend is expected to continue accelerating over the next century^17^. Between the years 2022–23, India recorded 3,27,449 dengue cases, with 394 deaths. In contrast, chikungunya cases recorded 11,778 cases. Karnataka alone had 11,446 dengue cases in 2023, which included 609 cases from the Udupi district. At the same time, 18 positive chikungunya cases were reported from Udupi during the same period^18^. These reports pose a public health concern due to their geographical spread and increase in disease burden. Despite these alarming figures, detailed, localised studies have not explored the impact of climatic factors and land use on *Aedes* mosquito distribution in this region.

Several factors contribute to the expansion of *Aedes*-borne infections, such as rapid urbanisation, the presence of vectors, population movement, travel and trade, waste management, and climate change, influencing the breeding and proliferation of vectors^7,8^. Various climatic factors and the changes in vectors, viruses, and hosts are dynamic. Temperature change causes a shift in disease incidence and prevalence, vector biting patterns, transmission frequency, and abundance. *Aedes* mosquitoes are highly adaptive to temperature variations. An increase in ambient temperature generally accelerates their development rate, shortens the extrinsic incubation period (EIP) of the virus, and can increase biting frequency. However, extreme temperatures may reduce adult mosquito longevity, making temperature both a facilitator and limiter of vector competence and virus transmission. Additionally, a change in humidity is believed to affect the development and lifecycle of *Aedes* larvae. Humidity is associated with the monsoon season, leading to smaller transmission cycles and rapid spread of infection. Searching and eliminating the containers during the monsoon season is necessary, as they can act as breeding habitats^8,19,20^.

Numerous research studies have utilised remote sensing (RS) and geographic information systems (GIS) to assess malaria susceptibility, delineate dengue risk zones, and analyse the dynamics of other VBDs across different periods^8,21^. Public health professionals and environmental scientists increasingly use these technologies to link urban land use with mosquito abundance and malaria prevalence, examining the close association between environmental changes, climate variables, and malaria epidemics. Several studies have shown that spatial and temporal risk factor mapping using surveillance data is crucial for preventing various VBDs like malaria and dengue^22^. GIS supports overlay and network analysis by documenting spatial parameters, giving epidemiologists access to extensive ecological and climatic data. Visualising epidemiological data in a geographic context and linking it with GIS software aids in prediction and risk profiling^8,22^. High spatial resolution satellite data offer detailed and real-time digital information as a basis for spatial baseline mapping. These data can be utilised periodically over several years to create precise land-use maps, essential for interpreting low-resolution satellite and socio-economic and geographical data. The choice of a satellite sensor with the appropriate spatial resolution is crucial for specific applications. Recent advancements in accessing remotely sensed vegetation and weather data, coupled with GIS, have opened new avenues for risk assessment, monitoring, and early warning systems^8,23,24^.

Mapping and predicting early disease outbreak warnings in vector surveillance systems are crucial. The WHO’s global dengue prevention plan emphasises developing predictive models to evaluate dengue outbreak risks. Understanding the spatial distribution of *A. aegypti* can aid in preventing not only dengue epidemics but also other diseases associated such as chikungunya and Zika outbreaks^25^. Regression analysis determines the relationship between variables, such as the number of reported cases and temperature. While commonly used in financial contexts, this method is invaluable in predicting factors influencing disease spread based on historical data and research. The primary advantage of regression analysis is its ability to forecast future events based on past trends and patterns^26–28^.

This study discusses the spatial distribution of *Aedes* mosquitoes in relation to climatic variables and land use/land cover (LULC) patterns in Udupi taluk. It describes the study area and entomological survey design, outlines the data sources and statistical methods used, presents the results of mosquito abundance and statistical modelling, and concludes with implications for targeted vector control using GIS and remote sensing tools.

## Methods

### Study area

**Fig. 1:**
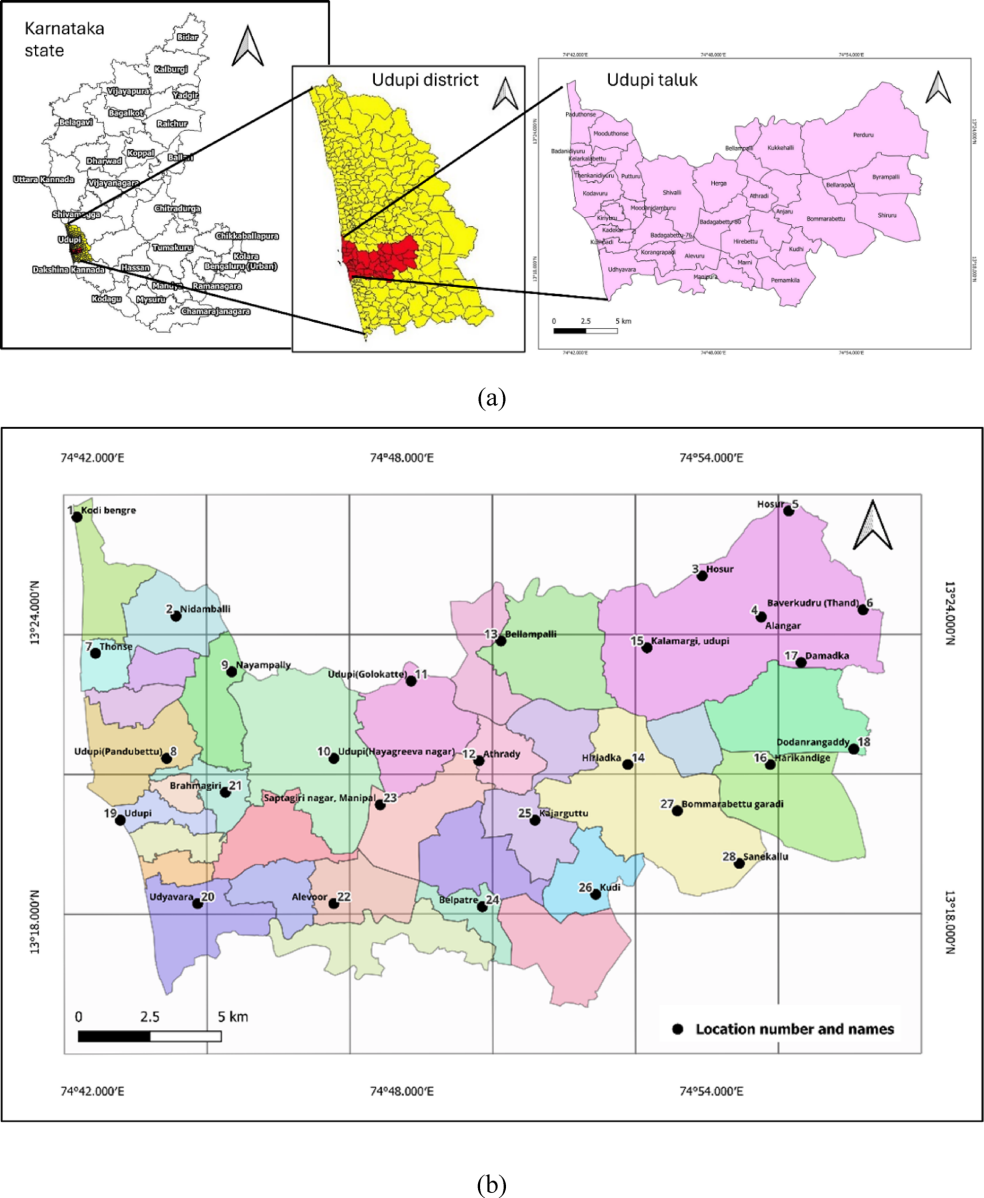
**(a) **Study area – Udupi taluk,** (b)** 5 × 5 km grid (Map and grid generated using QGIS Version 3.36.2, https://www.qgis.org).

Udupi taluk, located in the southwest region of South India in the Udupi district of Karnataka (Fig. 1a), is characterised by a tropical monsoon climate. It lies between latitudes 13.300°N and 13.550°N and longitudes 74.650°E and 74.850°E, covering an area of 924.13 km². According to the 2011 census, it has a population density of 818 people per km², with 58,086 households^29^. The region experiences warm temperatures year-round, ranging from 28 °C to 32 °C, and receives an average annual rainfall of approximately 4,119 mm. The high humidity level, averaging 78%, further defines its tropical monsoon climate^30^. This substantial rainfall and humidity create ideal breeding conditions for mosquito vectors, which pose significant public health challenges. The monsoon season, from June to September, is marked by heavy rainfall, which is crucial for agriculture but also influences the risk of vector-borne diseases due to the proliferation of mosquito breeding sites. In October and November, the post-monsoon season features warmer and damp conditions that provide optimal environments for *Aedes* mosquito breeding, necessitating heightened vector surveillance and control efforts. From December to February, winter brings cooler and drier conditions that reduce mosquito activity, though residual breeding sites may still pose public health risks. The summer season, from March to May, sees rising temperatures that reduce breeding sites due to evaporation, yet stagnant water from previous monsoons can sustain mosquito populations.

Udupi taluk features a mix of urban and rural landscapes, including residential areas, agricultural fields, forests, and water bodies. These diverse land use patterns influence mosquito distribution and abundance, impacting public health. In urban areas, dense populations and improper waste management can lead to water stagnation, providing breeding grounds for mosquitoes. Agricultural fields, with their irrigation practices, can create standing water that facilitates mosquito breeding.

Historically, Udupi taluk has faced challenges related to vector-borne diseases such as dengue, chikungunya, and malaria^31^. The high prevalence of *Aedes* mosquitoes, particularly during and after the monsoon season, underscores the need for targeted public health interventions. Public health initiatives in the region include regular monitoring of mosquito populations and breeding sites to identify and address potential outbreaks, community engagement to educate residents about preventive measures such as eliminating standing water and using mosquito repellents, and environmental management to reduce mosquito habitats.

Udupi taluk serves as a representative model for tropical monsoon regions facing similar public health challenges. The insights gained from this study can inform global strategies for managing *Aedes*-borne infections in tropical climates. Integrating Geographic Information System (GIS) and remote sensing technologies can be valuable for mapping environmental risk factors, detecting mosquito breeding habitats, and identifying high-risk areas thus enables public health authorities to prioritise resource allocation, such as deploying field teams for source reduction, targeting larvicide application, or planning awareness campaigns, in the most affected or high-risk areas, thereby enhancing the efficiency of vector control programs in regions like Udupi taluk. Given its unique climatic, geographic, and public health characteristics, Udupi taluk is an ideal study area for investigating the impact of climatic factors and LULC on mosquito populations and *Aedes*-borne disease transmission.

This cross-sectional, observational study examined the relationships between *Aedes* species abundance, environmental factors, and LULC variables during the post-monsoon period from October 2023 to December 2023. A 5 km X 5 km grid sampling technique was constructed using QGIS software version 3.36.2 to mark the sampling sites systematically. A total of 28 sampling locations were marked at each of the two diagonal corners of the grid cell, ensuring an even distribution across the study area (Fig. 1b).

The grid containing more than 50% of the geographical area belonging to the Udupi taluk was considered for marking the diagonal sampling locations, where each marked location was 2–3 km apart within the administrative boundary, as shown in Fig. 1b. The area belonging to the neighbouring taluk to the same grid was excluded from the study.

### House selection

At each sampling site, 20 houses were chosen for the survey, and a standard survey form was used to record geo-coordinates using the LatLong app, container details, and other specific attributes of mosquito breeding sites.

### Data collection

Entomological surveys and larval sampling were carried out between October 2023 and December 2023 to identify the distribution of mosquito fauna in the study region. The observation was made specifically in the sites where stagnant and small collected water originated from storing water for long periods or intermittent rainfall. The marked locations were visited, and the houses were screened for the presence of containers with stored water or discarded containers with stagnant water outside the housing premises. Larvae were collected using pipettes/cups/strainers in plastic containers and transported to the laboratory from all the breeding sites. The procedures and precautions regarding larval collection and transportation were according to the WHO and the National Vector Borne Disease Control Program (NVBDCP) guidelines^32^. The plastic containers with larvae samples were sealed tightly using mesh cloth and incubated at room temperature (25 °C to 30 °C) so that the larvae could develop into the adult stage. In the mature stage (adult mosquitoes), they were killed using ether. With the help of an aspirator tube, the killed mosquitoes were transferred to the test tubes to carry out morphological identification of species. The mosquito species were confirmed using a stereomicroscope based on the identification keys provided by the WHO^33^.

### Larval indices

Larval indices were estimated as the House Index (HI), Container Index (CI), and Breteau Index (BI)^34^.

$$\:\text{H}\text{o}\text{u}\text{s}\text{e}\:\text{I}\text{n}\text{d}\text{e}\text{x}\:\left(\text{H}\text{I}\right)\:=\left(\frac{\text{Number of houses infested}}{\text{Total number of houses inspected }}\right)\:X\:100$$

$$\:\text{C}\text{o}\text{n}\text{t}\text{a}\text{i}\text{n}\text{e}\text{r}\:\text{I}\text{n}\text{d}\text{e}\text{x}\:\left(\text{C}\text{I}\right)\:=\left(\frac{\text{Number of positive containers}}{\text{Total number of containers inspected}}\right)\:X\:100$$

$$\:\text{B}\text{r}\text{e}\text{t}\text{e}\text{a}\text{u}\:\text{I}\text{n}\text{d}\text{e}\text{x}\:\left(\text{B}\text{I}\right)\:=\left(\frac{\text{Number of positive containers}}{\text{Total number of houses inspected}}\right)\:X\:100$$

### Climatic data

Climate data were collected from meteorological stations for each sampling day at 28 locations. Data included average temperature in degrees Celsius and relative humidity in percentage obtained from the National Aeronautics and Space Administration Prediction of Worldwide Energy Resources (NASA POWER)^35^. The average rainfall (mm) was also obtained from the Indian Meteorological Department (IMD)^36^.

### Satellite image data

RS and GIS techniques were employed to analyse the influence of LULC on *Aedes* species distribution. High-resolution multispectral satellite imagery from Sentinel-2 (30 m spatial resolution) was obtained for the study area from Satellite images obtained from Bhuvan (by ISRO^37^, corresponding to the post-monsoon period (October–December 2023). The images were processed using QGIS version 3.36.2, and a supervised classification method (maximum likelihood algorithm) was applied to derive LULC categories. Training samples were collected through Google satellite reference data, visual interpretation, and ground-truth data from entomological survey sites. Classified land use/cover types included river, lake, forest, grassland, cropland, built-up areas, and barren land. Accuracy assessment was performed using an error matrix and kappa statistics based on randomly selected validation points. The final LULC map was overlaid with mosquito abundance data to identify spatial patterns and breeding hotspots.

Cohen’s Kappa coefficient.

$\:{\upkappa\:}\:=\frac{{P}_{0}-\:{P}_{e}}{1-{P}_{e}}$ where *P*_*o*_ ​ is the observed agreement,

*P*_*e*_​ is the expected chance of agreement.

**Fig. 2:**
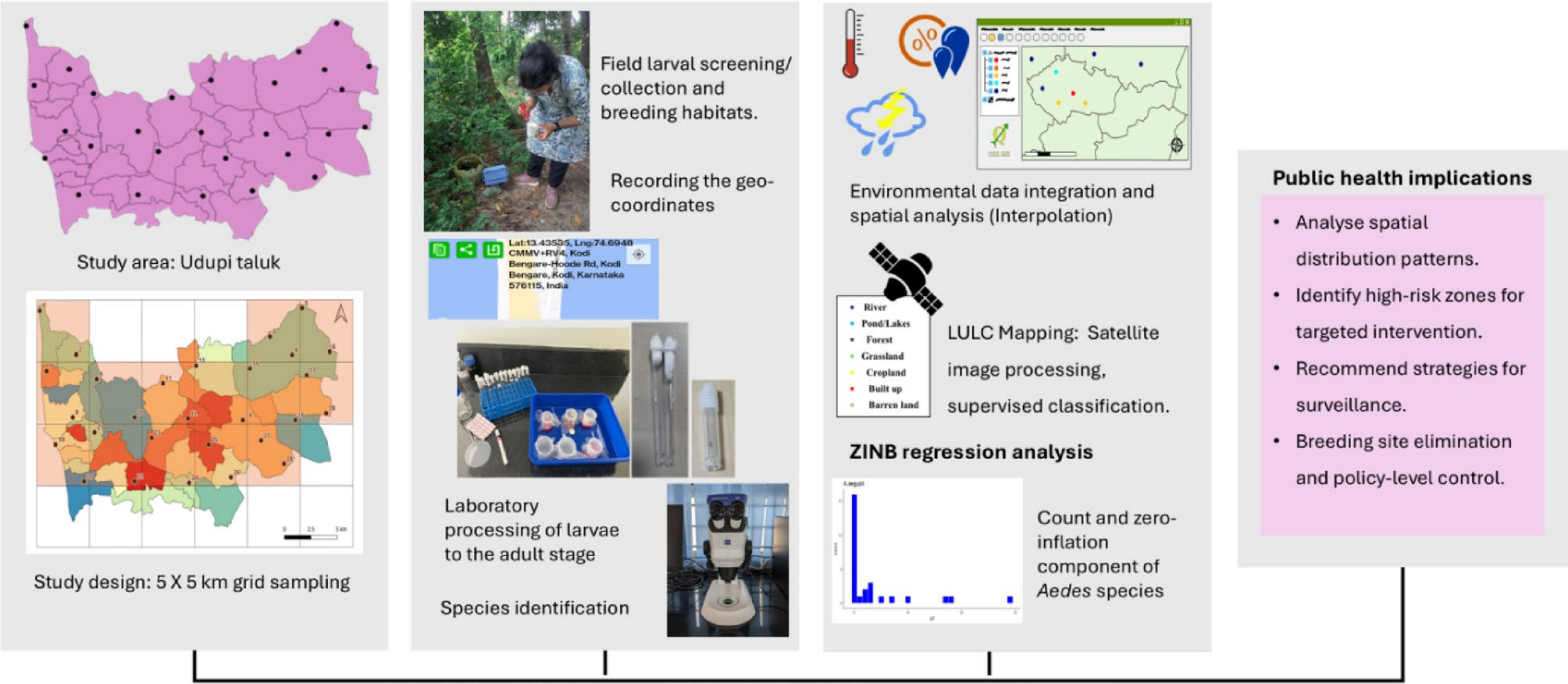
Methodology framework.

A stepwise methodological framework was followed to conduct the entomological and spatial analysis for *Aedes* species, as shown in Fig. 2.

### Spatial and statistical analysis

Spatial distribution of larval indices was analysed using heatmaps generated in QGIS (v3.36.2) through the Inverse Distance Weighting (IDW) technique, allowing visualisation of mosquito abundance patterns across the study area. In addition, IDW interpolation was also used to produce continuous spatial surfaces of *Aedes* larval density. IDW is a spatial interpolation technique used in GIS to estimate unknown values at a location based on known values from surrounding locations.

To classify mosquito breeding intensity across the 28 sampled locations, hotspot and coldspot representation was conducted using entomological survey data focused on *Aedes* breeding sites. Hotspots were defined as locations where *Aedes* larvae were detected in breeding habitats, indicating active breeding and higher transmission potential. Coldspots are referred to as locations where *Aedes* larvae were absent, but had conditions such as stagnant water, domestic containers, and the presence of other mosquito species. These hotspot and coldspot zones were overlaid on the LULC map to explore their association with various land use categories.

For statistical analysis, a Zero-Inflated Negative Binomial (ZINB) regression model was applied to investigate the relationship between *Aedes* mosquito counts and key predictors such as larval indices and environmental variables (e.g., temperature, humidity, rainfall). The model accounted for overdispersion and excess zeros in the count data. Model fit was assessed using the Akaike Information Criterion (AIC). All statistical analyses were performed using R software (version 4.4.3).

## Results

Out of 560 houses screened for larvae at 28 locations, 400 houses (71.42%) at 20 locations were found to have *Aedes* larval presence during the study period. The larval collection was focused on peridomestic habitats only, in close proximity to the households.

### Larval indices

**Fig. 3:**
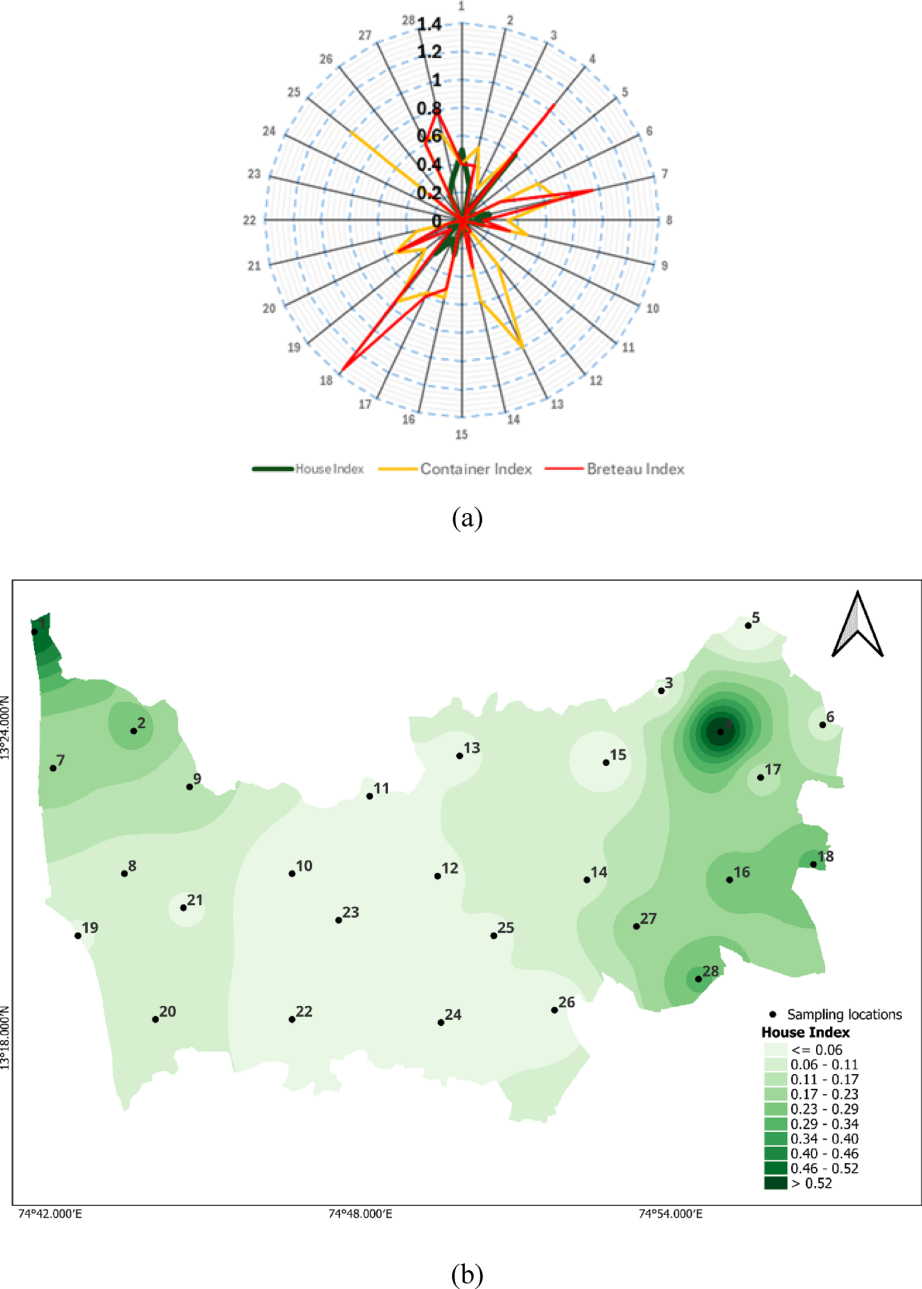

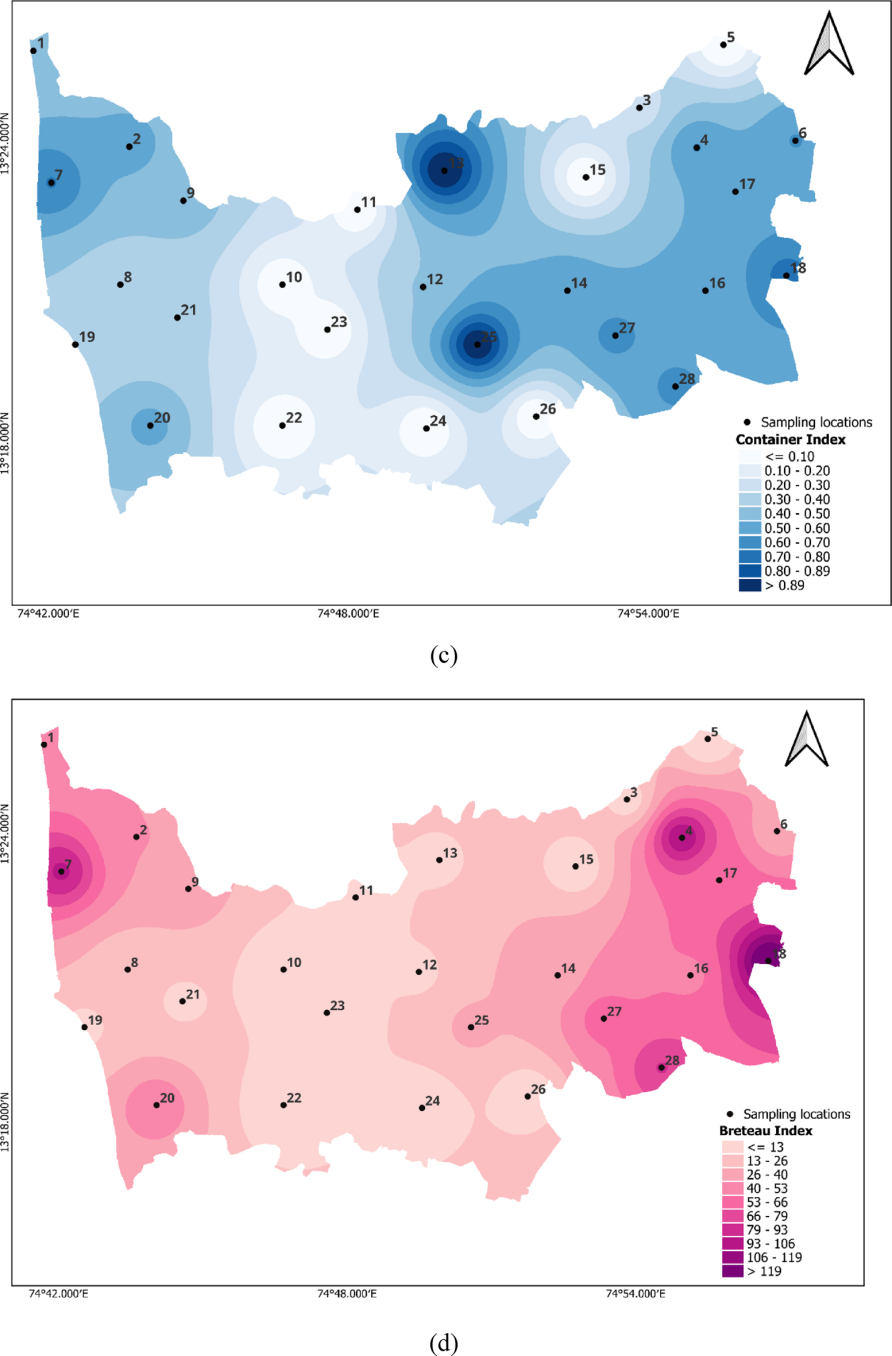
a. Radar chart showing the larval indices, **b**. House Index, **c**. Container Index, **d**. Breteau Index (Maps generated using QGIS Version 3.36.2, https://www.qgis.org).

The entomological indices recorded in this study, particularly the House Index (HI), Container Index (CI), and Breteau Index (BI), indicate a substantial risk of dengue virus transmission in several locations. According to WHO criteria, HI > 0.05, CI > 0.1, and BI > 20 are considered thresholds for heightened transmission potential. In our study, HI reached 0.6 in some locations, while CI reached 1, far exceeding these benchmarks. Although BI varied, it surpassed 20 in multiple sites. These elevated values, particularly in the post-monsoon period, align with conditions favourable to *Aedes* mosquito proliferation and point to a need for intensified vector control efforts and community-based source reduction strategies. Figure 3a Larval indices depict the larval indices of our study across 28 locations, showing high values with HI at 0.6% for location 4, CI at 1 for locations 13 and 25, and BI greater than 100 for locations 4 and 18. Figure 3b, c & d for HI, CI and BI illustrate the heatmaps for larval values observed at specific locations.

### Land use/land cover (LULC) classification

**Fig. 4:**
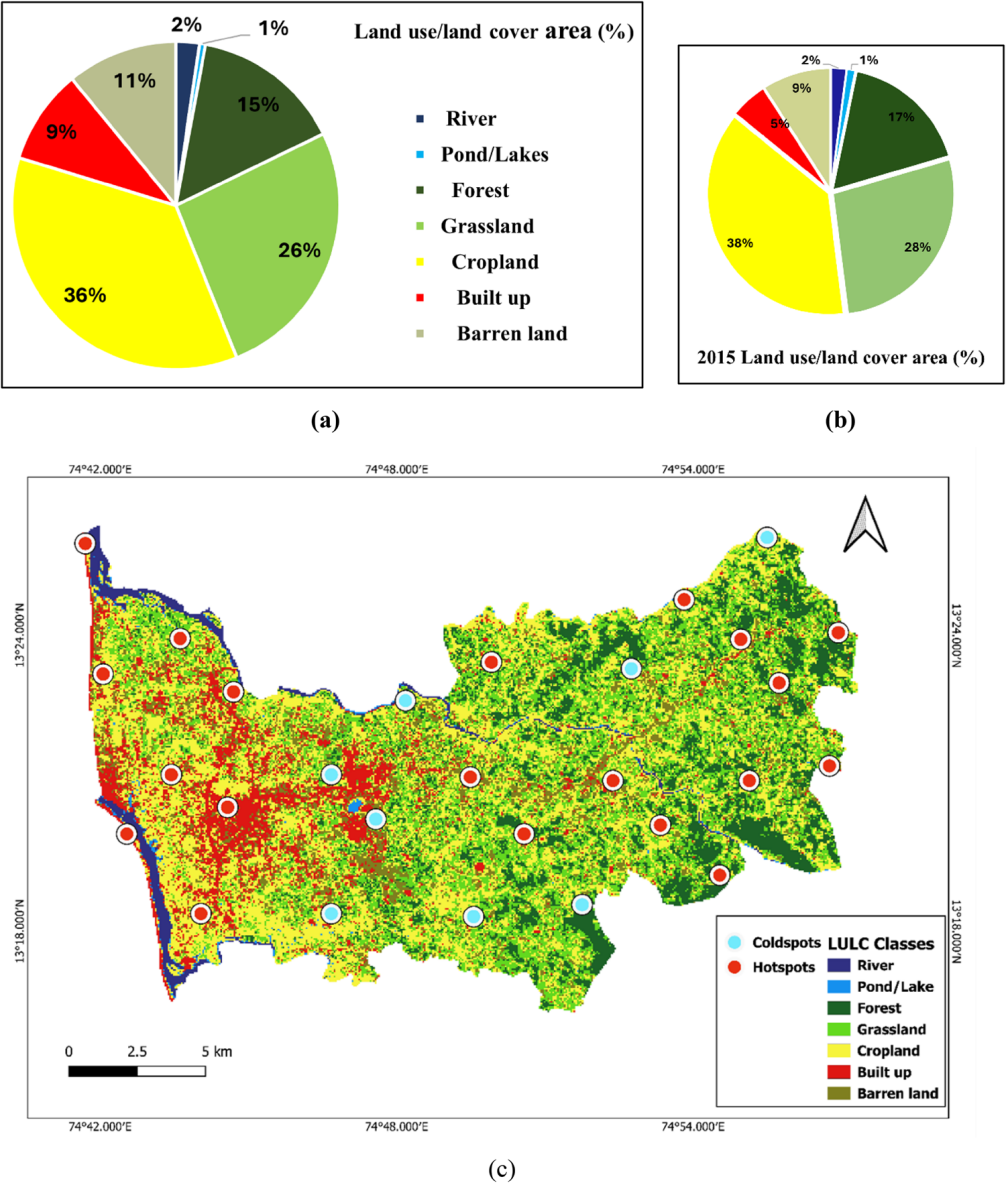
a. LULC classification year 2023, **b**. LULC classification year 2015, c. Hotspots and cold spots of *Aedes* breeding within the LULC map (LULC Map generated using QGIS Version 3.36.2, https://www.qgis.org).

The study area was classified by the supervised classification method and divided into seven classes, i.e., river, pond/lake, forest, grassland, cropland, built-up, and barren land. The error matrix observed an overall accuracy of 98.59%, evaluated based on Cohen’s Kappa coefficient. This Cohen’s kappa value indicates an ‘almost perfect’ agreement between the classified and reference data, suggesting that the classification process was accurate and reliable. Figure 4a and b show LULC maps of 2023 and 2015, respectively. The built-up area was 15.54 sq km (5%) to 29.36 sq km (9.36%) from 2015 to 2023 and also describes the percentage of land use change in each class.

### Hotspots and cold spots of *aedes* breeding within the LULC map

*Aedes* Larval breeding was detected in 71.43% of the surveyed locations within the study area. When these larval survey locations were overlaid on the LULC maps, it was observed that *Aedes* larval breeding habitats, i.e. hotspots, were predominantly (42.86%) located in fragmented urban landscapes and agricultural regions. The remaining 28.57% of the locations, which had non-*Aedes* mosquito breeding activity or absence of *Aedes* larval breeding, are found in other land cover classes such as forests and grasslands, but these locations had a conducive environment for larval breeding, such as stagnant water in containers, and other domestic mosquito species breeding were observed. Figure 4c hotspots and cold spots for *Aedes* species suggest favourable conditions for breeding, such as the availability of stagnant water or suitable microhabitats.

### *Aedes* species larva abundance

**Fig. 5:**
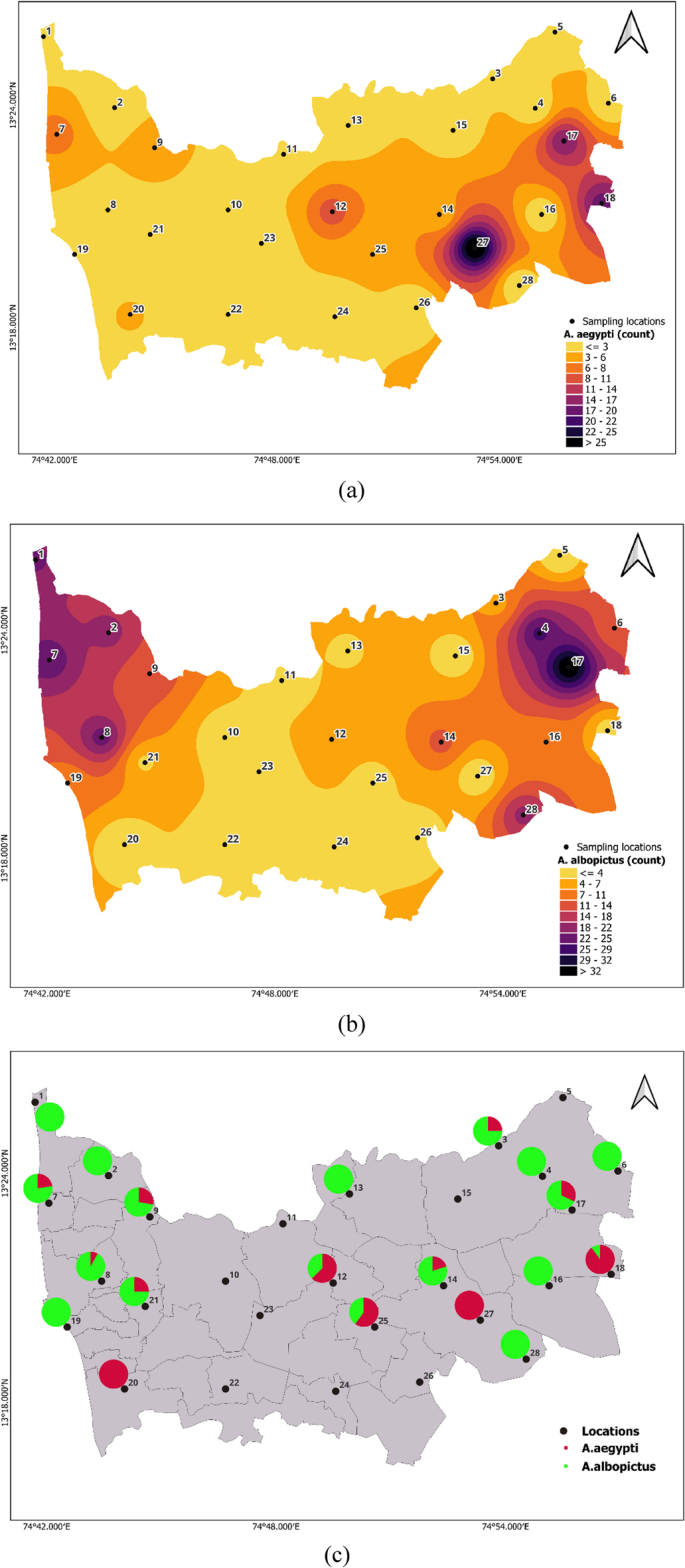
Larval density maps (**a**) *A. aegypti* (**b**) *A. albopictus (*Maps generated using QGIS Version 3.36.2, https://www.qgis.org).

A total of 425 mosquitoes were collected, among which *n* = 100 (23.52%) were *A. aegypti* and *n* = 245 (57.64%) were *A. albopictus*; the remaining mosquito collection belonged to the genus *Culex* and *Armigeres*. Through larval density mapping, a high abundance of *A. aegypti* was found at locations 17 (*n* = 17), 18 (*n* = 18), and 27(*n* = 29), whereas *A. albopictus* breeding was observed at locations 1 (*n* = 22),7 (*n* = 24),8 (*n* = 23),17(*n* = 36) and 28 (*n* = 19). As shown in Fig. 5 larval density (5a,b) and Aedes abundance (5c) across locations maps. Pie charts represent the relative abundance of A. aegypti and A. albopictus at each site (Fig. 5c). The figure highlights the predominance of A. albopictus across most localities, demonstrating spatial heterogeneity in species composition.

The survey found significant variability in environmental conditions across different locations, impacting the distribution of *Aedes* species at a temperature range of 23.4 °C to 26.3 °C, humidity of 70.1% to 88.5%, and intermittent rainfall of 0 to 61 mm. Under these conditions, the data indicated a higher prevalence of *A. albopictus* than *A. aegypti* across most surveyed locations (Fig. 5c).

### Larval breeding habitats

A wide range of artificial containers contributed to *Aedes* breeding across surveyed locations, reflecting variability in household water storage practices and waste management.

**Fig. 6:**
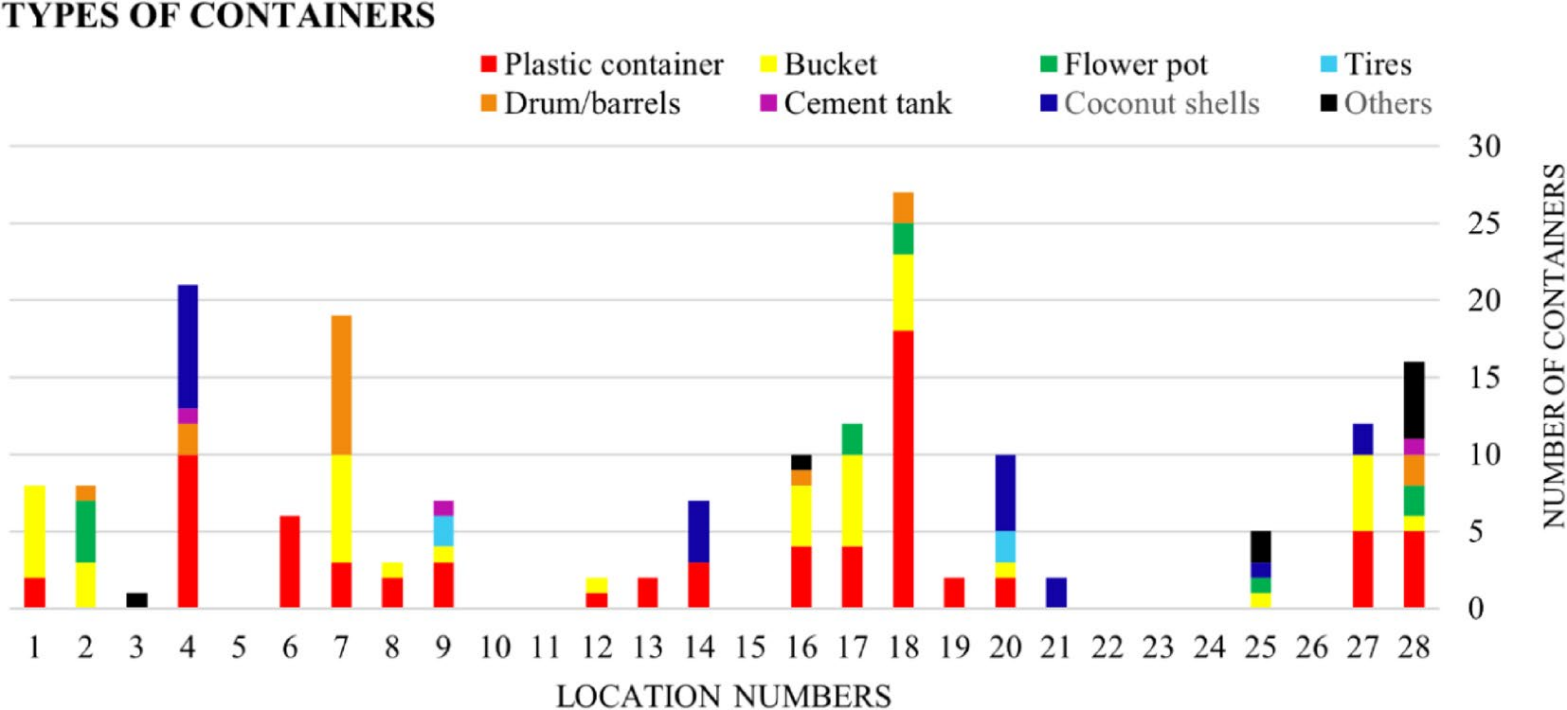
Number of various types of containers present in different study locations.

**Fig. 7:**
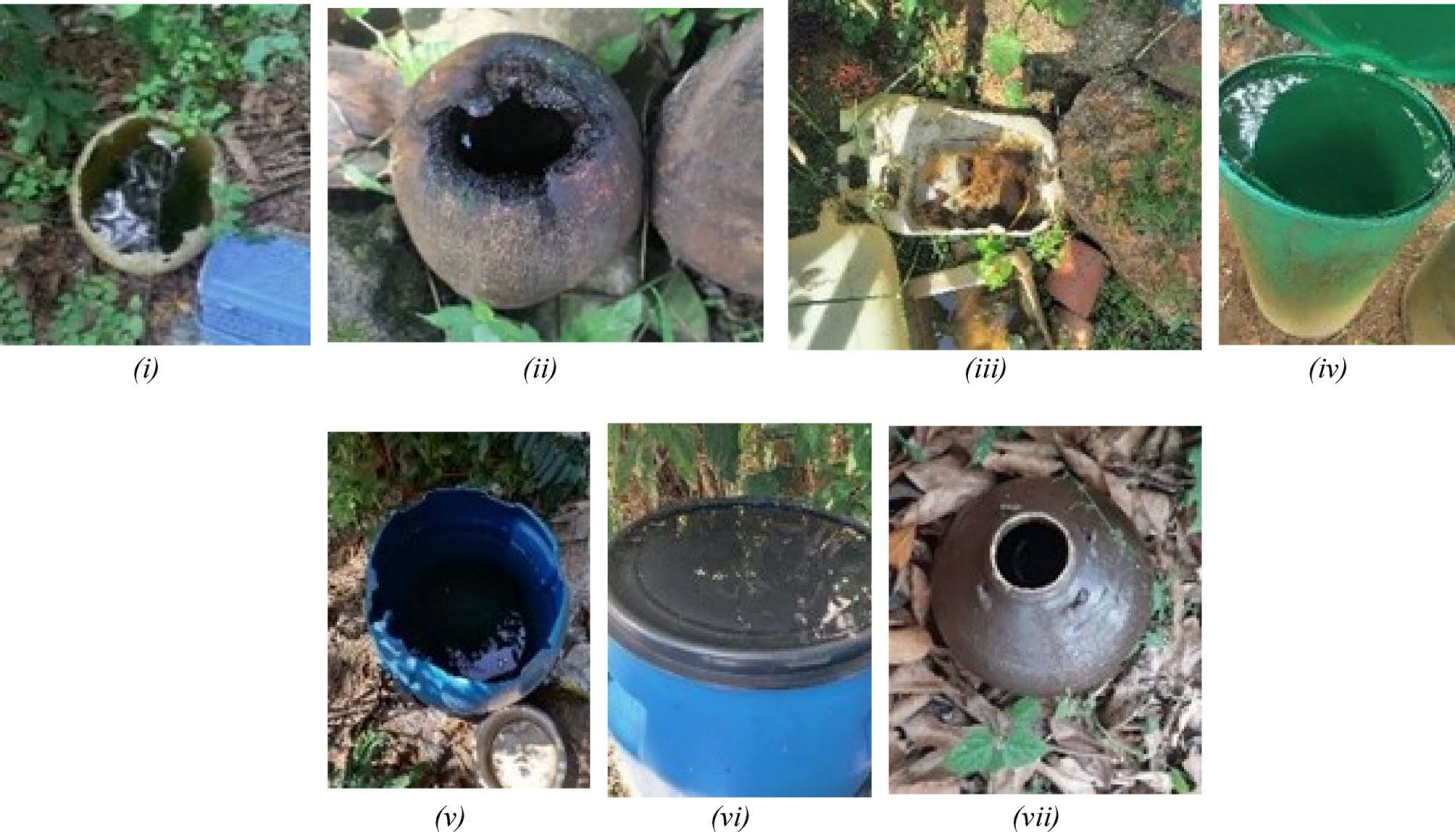
Breeding habitats: (i) Broken plastic container, (ii) Coconut shell, (iii) Broken plastic can, (iv) Drum, (v) Plastic container, (vi) Drum lid, (vii) Mud pot. (ii), (iii), (iv), (vi), (vii).

Figures 6 and 7 illustrates the different types of breeding containers observed during the survey; Location 18 (*n* = 27) shows a high number of plastic containers, location 4 (*n* = 21)observed multiple plastic containers, as well as coconut shells, and Location 28 (*n* = 16), had plastic containers, discarded broken plastic materials and plastic bags with depressions and grooves with stagnant.

### Statistical analysis

The impact of environmental factors (temperature, humidity, and rainfall) and larval indices (HI, CI, and BI) on the prevalence of the *Aedes* mosquito breeding habitats was assessed using Spearman correlation.

**Table 1:** Descriptives: median and Inter-quartile range of all variables.

	Temperature	Humidity	Rainfall	HI	CI	BI	A.aegypti	A.albopictus
N	28	28	28	28	28	28	28	28
Median	25.0	77.6	0.01	0.07	0.43	0.22	0.00	4.50
IQR	1.06	5.49	0.35	0.20	0.60	0.50	3.00	14.5
Shapiro-Wilk W	0.96	0.96	0.27	0.79	0.90	0.84	0.59	0.82
Shapiro-Wilk p	0.54	0.43	< 0.001	< 0.001	0.012	< 0.001	< 0.001	< 0.001

*p < 0.05 is considered significant, N is the no. of observations (sampling locations).

The normality of the data was assessed using the Shapiro-Wilk test. As shown in Table 1, temperature and humidity followed a normal distribution (*p* > 0.05), whereas HI, CI, BI, rainfall, and *A. aegypti/A. albopictus* counts deviated significantly from normality (*p* < 0.05). These results, further supported by Q-Q plots (Supplementary file, S1), guided the use of non-parametric methods, including Spearman correlation and Zero-Inflated Negative Binomial (ZINB) regression, for appropriate statistical analysis.

The correlation matrix highlights significant relationships shown in Table 2 between the HI, CI, and BI, which exhibit strong positive correlations. Temperature exhibits a moderate positive correlation with humidity and rainfall among the climatic variables. However, it shows weak or no significant correlations with the breeding indices and *Aedes* species counts. Meanwhile, humidity strongly correlates with rainfall but shows no significant correlation with *Aedes* species counts. While *A. aegypti* shows weak to moderate positive correlations with the breeding indices, it does not significantly correlate with environmental factors such as temperature, rainfall, and humidity. *A. albopictus* exhibits a strong positive correlation with the HI and medium correlations with the CI and BI, alongside a weak positive correlation with temperature.

**Table 2:** Correlation of Climatic variables and larval indices with *Aedes* species.

	HI	CI	BI	Temperature	Humidity	Rainfall	A. aegypti	A. albopictus
HI	-							
CI	0.735	-						
BI	0.948	0.830	-					
Temperature	0.181	−0.094	0.082	-				
Humidity	−0.071	−0.141	−0.112	0.241	-			
Rainfall	−0.155	−0.325	−0.228	0.388	0.705	-		
*A. aegypti*	0.332	0.472	0.497	−0.085	−0.114	−0.177	-	
*A. albopictus*	0.740	0.509	0.655	0.421	0.072	−0.014	0.195	-

*p < 0.05 is considered significant.

#### ZINB regression model for *A. aegypti* species

**Fig. 8:**
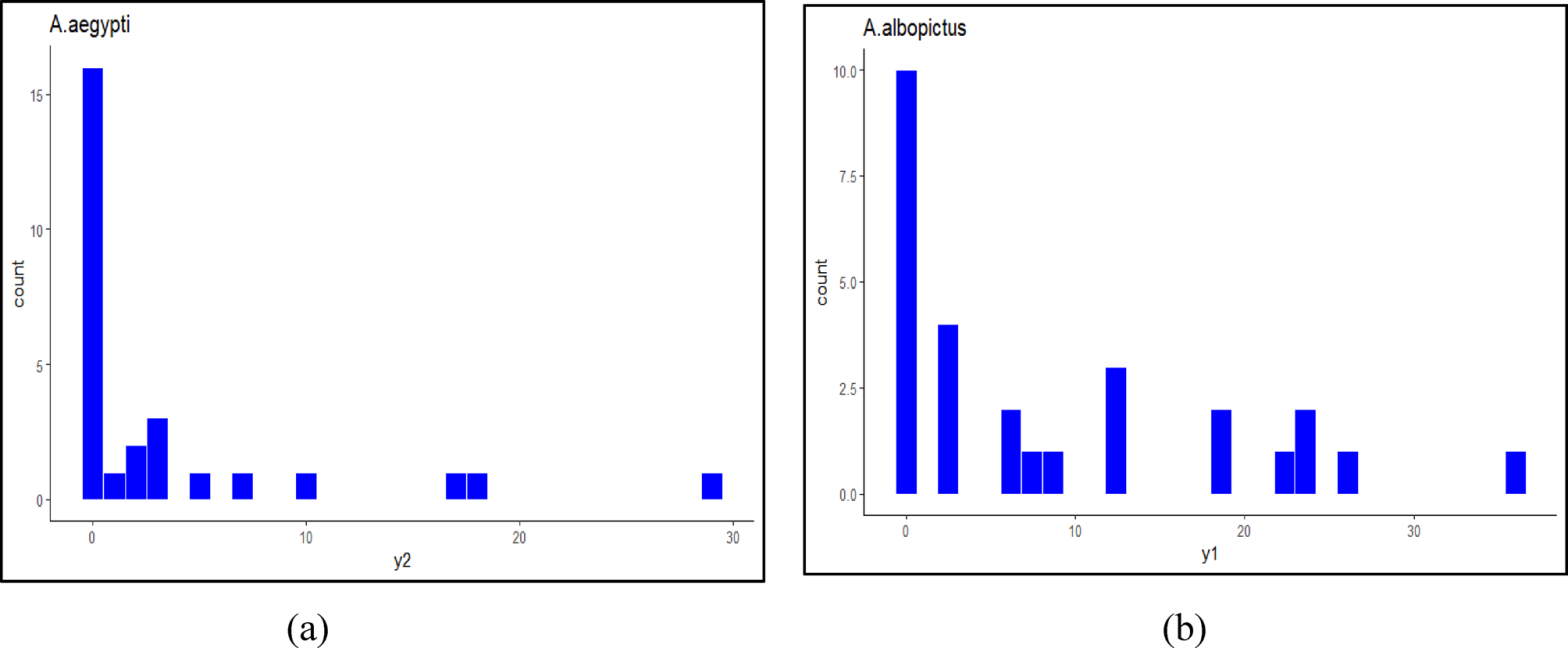
Frequency distribution of *Aedes* species (**a**) *aegypti*, (**b**) *A. albopictus*.

A stepwise regression identified HI and CI as significant predictors in the best-fit model, achieving an AIC of 94.17019. This approach ensures robust modelling, crucial for understanding the factors influencing mosquito populations and disease transmission dynamics. The ZINB regression model was used to analyse the influence of larval indices on the abundance of *A. aegypti*. The results from the count component of the model (Fig. 8; Table 3) indicate that a one-unit increase in the HI is associated with a statistically significant increase in the log count of *A. aegypti* by approximately 11.56 **(***p* < 0.05**)**, after adjusting for the CI. This suggests that areas with higher household-level larval positivity are likely to have greater *A. aegypti* abundance. In contrast, the CI did not show a statistically significant association with *A. aegypti* abundance in the count model (*p* > 0.05), indicating that the proportion of infested containers, relative to all inspected water-holding containers, may not directly influence the mosquito abundance.

In the zero-inflation component of the model, HI was associated with an odds ratio of 305.85 for an observation being an excess zero (i.e., no larvae despite favourable conditions). However, this effect was not statistically significant **(***p* > 0.05), implying that HI does not reliably predict the likelihood of zero counts due to structural zeros. Similarly, the effect of CI in the zero-inflation model was also non-significant (*p* > 0.05). Overall, the model highlights HI as a key predictor of *A. aegypti* abundance, while the CI appears to have limited explanatory power in both the count and zero-inflation components.

**Table 3:** ZINB regression model for *A. aegypti*.

Predictor For A.aegypti	Coefficient	Standard error	z-value	*p*-value	95% confidence interval (Lower: 2.5%)	95% confidence interval (Upper: 97.5%)
Count model						
Intercept	0.620	0.827	0.750	0.453	−1.001	2.243
HI	11.555	3.787	3.057	0.002	4.133	18.978
CI	−0.6451	1.408	−0.458	0.647	-	-
Zero-inflation model						
Intercept	13.46	42.18	0.319	0.750	−61.292	96.122
HI	305.85	487.74	0.627	0.531	−650.105	1261.797
CI	−151.66	248.93	−0.609	0.542	-	-

*p < 0.05 is considered significant.

#### ZINB regression model for *A. albopictus* species

Stepwise regression identified the HI as the best predictor for *A. albopictus* breeding, with the selected model yielding the lowest AIC (148.88). However, as shown in Table 4; Fig. 8, the count model revealed that although HI had a positive coefficient (β = 2.33), this effect was not statistically significant (*p* = 0.109). The wide 95% confidence interval (–0.52 to 5.17) further reflects uncertainty in the estimate.

Similarly, the zero-inflation component of the model indicated that HI did not significantly influence the probability of excess zeros (β = − 261.56, *p* = 0.365), suggesting that HI alone may not explain the absence of *A. albopictus* in some areas. This lack of statistical significance may reflect the ecological complexity of *A. albopictus*, which is known to occupy a wide range of habitats and respond to multiple environmental factors not fully captured in this model.

**Table 4:** ZINB regression model for *A. albopictus*.

Predictor For A. albopictus	Coefficient	Standard error	z-value	*p*-value	95% confidence interval (Lower: 2.5%)	95% confidence interval (Upper: 97.5%)
Count model						
Intercept	2.025	0.335	6.048	1.470	1.369	2.682
HI	2.3281	1.457	1.603	0.109	−0.517	5.173
Zero-inflation model						
Intercept	6.625	10.114	0.655	0.512	−13.198	26.449
HI	−261.561	288.719	−0.906	0.365	−827.43	304.317

*p < 0.05 is considered significant.

## Discussion

This study aimed to investigate the spatial distribution and ecological determinants of *Aedes* mosquito breeding in Udupi taluk using entomological surveys, larval indices, and a statistical modelling approach.

## Entomological indices and vector risk

The HI, CI, and BI are crucial indicators for assessing the risk of dengue virus transmission. In our study, BI exceeded this threshold in several locations due to the presence of multiple positive containers per household, indicating intense breeding activity^38^. These elevated indices underscore a heightened risk for arboviral transmission and the urgent need for targeted vector control. Findings of an entomological survey conducted in the state’s capital city corroborate our findings, as they found significant *Aedes* breeding containers in the peridomestic and intra-domestic habitats, reported a high HI, CI and BI of 0.2, 0.3 and 0.5, respectively, during the pre-monsoon time. Since that study was conducted in collaboration with the state’s health department, it was feasible for them to explore the intra-domestic habitats^13^. The presence of *Aedes* larvae in the peridomestic habitats of our study locations indirectly indicates their presence even inside the households. It is well-known that the elimination of breeding sites is the most important step in curbing mosquito-borne diseases. In this context, state-wide entomological surveillance in collaboration with public health authorities could be a timely and effective step, particularly in regions with a high disease burden.

## Spatial patterns

IDW interpolation techniques revealed distinct spatial patterns of larval indices and mosquito abundance. The spatial heat maps of larval indices highlight the areas where targeted mosquito control efforts and public health interventions may be needed to reduce the risk of mosquito-borne diseases and protect the community’s health^39^. Few studies have explored the integration of satellite data for LULC in relation to *Aedes* proliferation, which can guide towards the preventive, timely measures towards the growing urbanisation and population^40–42^. LULC classification data from 2015 to 2023 revealed a notable increase in built-up areas, which corresponded spatially with identified *Aedes* hotspot zones, suggesting that rapid urban expansion may be a key driver of enhanced vector breeding potential and dengue transmission risk. *Aedes* breeding hotspot areas were concentrated in fragmented urban landscapes and croplands. In this study, *Aedes* breeding hotspots were identified in areas with active larval presence of *Aedes* species, indicating high transmission potential, while coldspots lacked *Aedes* larvae but exhibited conducive conditions for future. This highlights the importance of monitoring even the low-risk areas for proactive vector control. A similar study in Maharashtra’s Konkan region linked disease risk zones with rapid urbanisation and land-use changes, aligning with our findings that fragmented and poorly managed landscapes support mosquito proliferation^43^. This spatial distribution highlights the need for customised intervention strategies based on specific land use patterns.

### Climatic influence

The environmental conditions, particularly temperature and humidity, along with prior trends (Table 5), appear to play crucial roles in the distribution of these species^44,45^. In this study, temperature and humidity appeared to influence *Aedes* distribution, although not all associations reached statistical significance. According to the WHO, if the annual rainfall exceeds 1500 mm, that region is categorised as a tropical region. As per the previous five-year annual rainfall data (Table 5), the Udupi region receives an average of 4119 mm of rain, which is much higher, and hence, it’s a tropical monsoon zone, an ideal ecological niche for vector breeding^46^. Several studies have shown that higher rainfall is associated with vector abundance^47,48^. While this study did not find significant correlations between vector abundance and environmental variables such as rainfall and humidity, these factors may still play an influential role, and this deserves further investigation.

**Table 5:** Prior trends of environmental variables.

Year	Average Temperature (^0^C)	Relative Humidity (%)	Actual Rainfall (mm)
2022	25.14	80.01	4253.37
2021	25.39	80.31	4459.89
2020	25.70	78.69	4773.79
2019	26.13	74.57	4563.11
2018	25.64	74.46	4360.81

## Breeding habitat and anthropogenic activity

A similar study was conducted on 500 household surveys in Udupi taluk from March to August 2012, where the foremost factors for *Aedes* breeding were coconut shells and uncovered water containers^46^. This study of the post-monsoon survey revealed a wider diversity of habitats, including plastic buckets, cement tanks, disused tyres, and waste plastic items^46^. This broader spectrum may reflect environmental and behavioural shifts over the past decade, such as increased urban development, poor waste management, and seasonal differences in water^14^. These findings highlight the evolving nature of vector breeding ecology in response to changing land use and human practices.

## ZINB regression analysis

Traditional count models were insufficient due to excess zero values in the dataset, prompting the use of ZINB regression^49^. For *A. aegypti*, HI was a significant positive predictor in the count component, supporting its use as a surveillance tool. However, neither HI nor CI were a significant predictor in the zero-inflation model, suggesting that other unmeasured factors may influence the presence of excess zeros. In contrast, *A. albopictus* showed no statistically significant associations in the count or zero-inflation components, though HI emerged as the best fit model based on AIC. This may be attributed to the species’ broader ecological tolerance and the influence of unaccounted environmental factors such as vegetation, microclimate, and container types. Similar findings from Florida and Southeast Asia indicate that *A. albopictus* populations often display unpredictable spatial patterns, highlighting the need for expanded variables such as vegetation cover, shade, socio-economic details and microclimatic conditions in future models^28,50–52^.

## Implications for public health and vector control

This study reinforces the critical role of household-level source reduction in *Aedes* mosquito control. The detection of larvae in peridomestic areas likely reflects breeding activity within intra-domestic spaces, which were not sampled due to access limitations. This underscores the need for comprehensive surveillance encompassing both indoor and outdoor environments. The integration of GIS and RS proved effective for identifying larval hotspots and informing spatially targeted interventions. These tools can enhance the precision of vector control efforts by aligning them with high-risk zones derived from entomological and environmental data. Collaboration with public health authorities is essential, particularly to facilitate access to intra-domestic sites and strengthen entomological surveillance. Expanding the temporal coverage of sampling across multiple seasons and incorporating dengue case data would allow for a more complete understanding of transmission dynamics and seasonality. The observed association between *Aedes* abundance, climatic variables, and LULC highlights the complex ecological drivers of mosquito proliferation.

The findings provide a basis for evidence-based policymaking aimed at mitigating dengue transmission. Effective strategies may include community engagement for source reduction, enhancing water management practices, and leveraging climate forecasts for proactive vector control. This multi-pronged approach can enhance the effectiveness of dengue control programs and reduce the disease burden in endemic regions like Udupi.

## Conclusion

This study highlights the influence of climatic variables and LULC patterns on *Aedes* mosquito distribution during the post-monsoon period in Udupi taluk. The integration of GIS and RS techniques enabled spatial analysis of larval indices and environmental risk factors, identifying breeding hotspots for targeted vector control. Findings support the use of spatial decision tools in *Aedes* surveillance programs to enhance vector management strategies.

## Limitations

- The study was limited to a post-monsoon surveillance period (October–December 2023), which may not capture seasonal variations in vector dynamics.
- Entomological sampling was limited to one Taluk of a district; also, a larger spatial coverage with a larger sample size would increase the statistical reliability of findings. Only climatic and land-use factors were included; other variables such as socio-economic conditions, water management practices, and human movement were not assessed.
- This study lacks a quantitative measurement of the distance between identified breeding sites and human dwellings. While all breeding containers were found within residential premises, explicitly mapping their spatial proximity could have provided a more nuanced understanding of transmission risk, considering the limited flight range of *Aedes* mosquitoes.

## Future directions

- Conducting longitudinal studies across multiple seasons to assess temporal variations in *Aedes* abundance and transmission risk.
- Integrating socio-economic and behavioural data to improve predictive modelling.
- Explore the application of machine learning algorithms for real-time outbreak forecasting with the integration of dengue case data.
- Evaluate intervention outcomes using spatial risk maps to inform adaptive control strategies in high-risk zones.

## Supplementary Information

Below is the link to the electronic supplementary material.

Supplementary Material 1

## Data Availability

All relevant data are included in the paper.
