# Supplementary material for: Influence of climatic and land use factors on post-monsoon distribution of Aedes mosquito vectors in Udupi taluk
Source: Sci Rep. 2025 Oct 21;15:36649. doi: 10.1038/s41598-025-20413-y (PMC12540794; doi:10.1038/s41598-025-20413-y)
Supplement: Supplementary file 1 — Supplementary Material 1 [file 41598_2025_20413_MOESM1_ESM.docx]

**Influence of climatic and land use factors on post-monsoon distribution of *Aedes* mosquito vectors in Udupi taluk**

**Prathiksha Prakash Nayak^1^, Jagadeesha Pai B^2*^, Sreejith Govindan^3*^, Naren Babu N^4^**

^1^ Research scholar, Department of Civil Engineering, Manipal Institute of Technology, Manipal Academy of Higher Education, Manipal, India. [prathiksha.nayak@learner.manipal.edu](mailto:prathiksha.nayak@learner.manipal.edu)

^2*^ Associate Professor, Department of Civil Engineering, Manipal Institute of Technology, Manipal Academy of Higher Education, Manipal, India. [jaga.pai@manipal.edu](mailto:jaga.pai@manipal.edu)

^3*^ Associate Professor, Division of Microbiology, Department of Basic Medical Sciences, Manipal Academy of Higher Education, Manipal, India. [g.sreejith@manipal.edu](mailto:g.sreejith@manipal.edu)

^4^ Lecturer, Manipal Institute of Virology, Manipal Academy of Higher Education, Manipal, India. [naren.babu@manipal.edu](mailto:naren.babu@manipal.edu)

***Corresponding author Email:** Jagadeesha Pai B, Sreejith Govindan

| 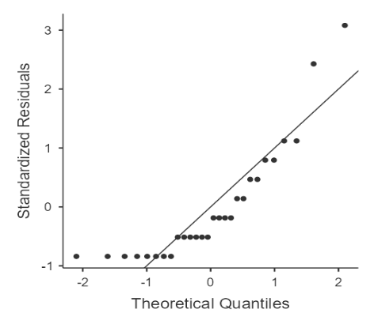 | 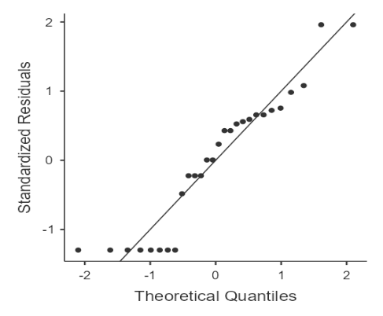 | | 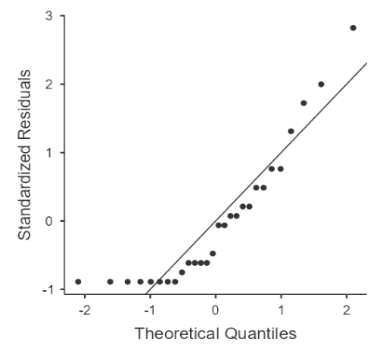 | |
| --- | --- | --- | --- | --- |
| (a) | (b) | | (c) | |
| 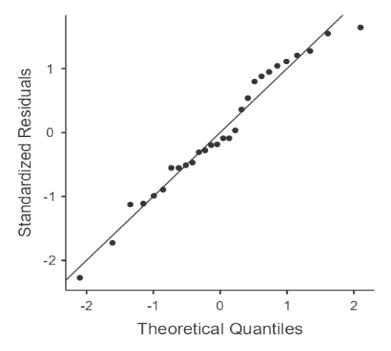 | 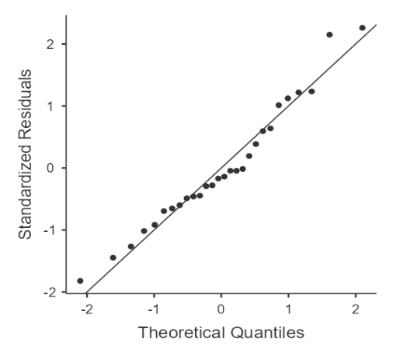 | | 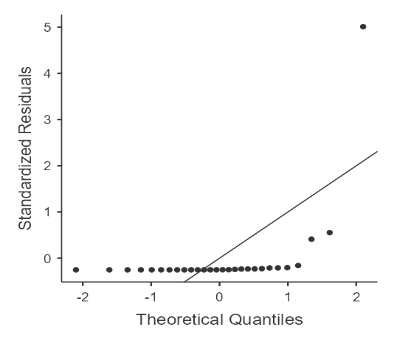 | |
| (d) | (e) | | (f) | |
| 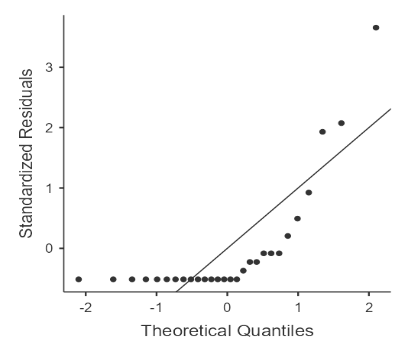 | | 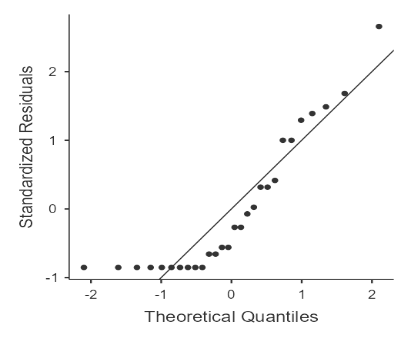 | |  |
| (g) | | (h) | |  |

**Supplementary Figure 1 (S1). Q-Q plots: (a) House index, (b) Container index, (c) Breteau index, (d) Temperature, (e) Humidity, (f) Rainfall, (g) Aedes aegypti, (h) Aedes albopictus**
